# Stratum-specific serum metabolic reprogramming in Saanen goats with graded Brucella serological reactivity under natural exposure

**DOI:** 10.3389/fvets.2026.1866534

**Published:** 2026-06-26

**Authors:** Xiaotong Zhao, Hua Chang, Jiming Li, Zhidong Zhang, Yongping Hu, Xun Xiang

**Affiliations:** 1 Laboratory of Animal Science and Technology, College of Animal Science and Technology, Yunnan Agricultural University, Kunming, Yunnan, China; 2 Laboratory of Veterinary Medicine, College of Veterinary Medicine, Yunnan Agricultural University, Kunming, Yunnan, China; 3 Animal Disease Prevention and Control Center of Shilin Yi Autonomous County, Kunming, Yunnan, China; 4 Chia Tai (Kunming) Co., Ltd., Kunming, China; 5 Feed Supervision and Inspection Institute of Honghe Prefecture, Mengzi, China

**Keywords:** *Brucella melitensis*, brucellosis, goat, LC–MS/MS, serological stratification, zoonosis

## Abstract

Goat brucellosis, caused by *Brucella melitensis*, is a globally important zoonosis, yet host metabolic responses across serological reactivity levels under natural field conditions remain poorly understood. In this exploratory cross-sectional study, 150 Saanen goats were stratified by two-step serology into seronegative (Group A), weakly seropositive (Group B; serum agglutination test (SAT) titer 1:50), and strongly seropositive (Group C; SAT titer ≥1:100) groups; eight animals per group (*n =* 24 total) underwent broad-targeted liquid chromatography–tandem mass spectrometry (LC–MS/MS) serum metabolomics, yielding 699 annotated metabolites across 39 categories. Metabolic separation intensified with serological reactivity and was most pronounced between Groups A and C. Amino acid metabolism pathways were consistently enriched across all pairwise comparisons, while pyruvate metabolism, the pentose phosphate pathway, and purine/pyrimidine metabolism showed stage-associated enrichment only in comparisons involving Group C. D-erythrose 4-phosphate was the sole metabolite significantly altered in all three pairwise comparisons, with monotonically increasing abundance (area under the curve (AUC): A vs. B = 0.766; A vs. C = 1.000; B vs. C = 0.891). Trimethylamine N-oxide exhibited a biphasic pattern, while symmetric dimethylarginine declined monotonically. Collectively, these results reveal a progressive, stratum-dependent reorganization of the serum metabolome in goat brucellosis and highlight D-erythrose 4-phosphate and several co-altered metabolites as priorities for validation in future studies.

## 1 Introduction

Goat brucellosis, caused by *Brucella melitensis*, is one of the most widespread zoonoses worldwide and the predominant source of human brucellosis (1–3). Since the domestication of goats, the risk of human *B. melitensis* infection has continued to expand, with approximately half of the global population currently at risk of exposure (1–3). Recent estimates place the global annual incidence of human brucellosis at approximately 2.1 million cases, with Africa and Asia bearing the greatest disease burden (4). In livestock, the disease manifests primarily as abortion, infertility, and markedly reduced milk yield, whereas in humans it presents with recurrent fever, osteoarticular complications, and obstetric sequelae, imposing substantial public health and economic consequences (5). Understanding how Brucella infection reshapes host metabolism may therefore open new avenues for biomarker discovery and more nuanced serological classification of infected animals.

Metabolomics, through the systematic quantification of dynamic changes in low-molecular-weight metabolites, directly reflects the terminal metabolic phenotype of the host following infection and represents a powerful tool for biomarker discovery and mechanistic investigation of host–pathogen interactions in infectious diseases (6, 7). Liquid chromatography–tandem mass spectrometry (LC–MS/MS)-based broad-targeted platforms can cover hundreds to thousands of metabolites in a single run, making them well suited to capturing the systemic metabolic shifts triggered by intracellular pathogens such as Brucella (8). Previous studies have demonstrated that Brucella infection induces a Warburg-like metabolic shift in host cells and upregulates host purine nucleotide *de novo* biosynthesis to support intracellular bacterial replication—infection-associated metabolic signatures amenable to detection by serum metabolomics (9, 10). In the broader context of animal disease research, metabolomics has shown considerable potential for biomarker identification, metabolic pathway characterization, and phenotypic stratification in infectious and inflammatory conditions (7, 11).

Although initial progress has been made in the metabolomic investigation of Brucella infection, significant limitations remain in the existing literature. Studies to date have predominantly adopted a binary case–control design comparing infected individuals with healthy controls. A serum metabolomics study employing ultra-high-performance liquid chromatography coupled with quadrupole Orbitrap tandem mass spectrometry (UHPLC-Q Exactive-Orbitrap MS/MS) identified differentially abundant metabolites in acute human brucellosis, principally associated with bile acid synthesis and purine metabolism (12). Broad-targeted serum metabolomics has further revealed marked perturbations in amino acid metabolism, the urea cycle, the tricarboxylic acid (TCA) cycle, and fatty acid metabolism in brucellosis patients (13). In an animal model, metabolic alterations in *B. melitensis*-infected murine tissues were found to be time-dependent, most pronounced during peak inflammation, and primarily affecting *γ*-aminobutyric acid (GABA) shunting and glutamine metabolism (14). However, the existing literature has not addressed the differential metabolic profiles associated with varying levels of serological reactivity under natural field conditions, nor has it systematically characterized host metabolic changes between weakly and strongly seropositive states (15). Moreover, the recognized limitations of current serological diagnostic methods with respect to sensitivity, specificity, and result interpretation underscore the need to explore complementary metabolic phenotype-based approaches to serological stratification (15). Critically, metabolomics investigations conducted under natural infection surveillance conditions are fundamentally distinct from studies employing experimental challenge models, in which infection dose, inoculation route, timing, and host background can be rigorously standardized. Under field conditions, serum metabolic profiles integrate the full complexity of the host–pathogen interaction as it unfolds across variable individual physiological backgrounds, representing a more epidemiologically relevant—but inherently more heterogeneous—biological context than controlled laboratory infection. To date, no study has characterized serum metabolic profiles across graded serological reactivity strata in naturally exposed goats.

To address these knowledge gaps, the present study enrolled Saanen goats under a natural infection surveillance context and stratified them into seronegative, weakly seropositive, and strongly seropositive groups according to serological reactivity. A broad-targeted LC–MS/MS metabolomics platform was applied to serum samples to characterize the metabolic profiles associated with distinct serological strata, to identify candidate metabolites with potential discriminant value, and to provide exploratory metabolomic evidence advancing the understanding of host metabolic responses to *B. melitensis* infection in goats.

## 2 Materials and methods

### 2.1 Ethics statement

All experiments involving animals were conducted in accordance with the “Guidelines for the Care and Use of Laboratory Animals” and conformed to the guiding principles of the Life Sciences Ethics Committee of Yunnan Agricultural University (approval no: 202604011).

### 2.2 Animal subjects and sample collection

This study was a cross-sectional metabolomics investigation conducted within a natural infection surveillance context, designed to compare serum metabolic profiles across Saanen goats exhibiting different levels of serological reactivity to Brucella. All blood samples were sourced from a Saanen goat breeding facility in Shilin Yi Autonomous County, Kunming, Yunnan Province, China, and were submitted to the Animal Disease Prevention and Control Center of Shilin Yi Autonomous County as part of routine brucellosis surveillance. All animals were maintained under standard husbandry conditions at the time of sampling. Whole blood was collected from each animal by trained veterinary technicians via jugular venipuncture; approximately 5 mL was drawn per animal into sterile centrifuge tubes. Samples were allowed to clot at room temperature (20–25 °C) for 30 min and then centrifuged at 4000 r/min for 5 min. The separated serum was transferred to sterile cryovials, assigned unique identifiers, and stored at −80 °C until serological testing and metabolomic analysis. During the metabolomics sample selection phase, samples of adequate quality with complete individual records were prioritized; samples exhibiting severe hemolysis, insufficient serum volume (< 200 μL), or evidence of concurrent disease—defined operationally as rectal temperature > 39.5 °C at the time of sampling, visible clinical signs (lameness, respiratory distress, diarrhea, or visible abscess formation), or confirmed serological reactivity to foot-and-mouth disease or caprine arthritis-encephalitis virus on concurrent routine surveillance—were excluded where applicable. As samples were derived from a natural surveillance context, residual confounders—including farm origin, dietary composition, and physiological status—could not be fully controlled; these factors are accounted for accordingly in interpreting the results.

### 2.3 Serological testing and stratification

#### 2.3.1 Rose Bengal plate agglutination test

All submitted serum samples were screened for brucellosis using a Rose Bengal plate agglutination test (RBPT) antigen (Qingdao Lijian Biological Technology Co., Ltd., Qingdao, China; veterinary drug approval number: 兽药生字154028017) (16, 17). Prior to testing, both the antigen and test sera were equilibrated to room temperature (20–25 °C) for 30–60 min. A volume of 0.03 mL of test serum was thoroughly mixed with an equal volume (0.03 mL) of antigen on a clean glass slide, rocked horizontally, and examined under natural light within 4 min for agglutination. Results showing agglutination of “+” or greater were classified as positive; uniformly turbid mixtures without visible agglutination (“−”) were classified as negative. All RBPT-positive samples were subsequently subjected to the serum agglutination test (SAT) for quantitative titration.

#### 2.3.2 Serum agglutination

RBPT-positive sera were quantitatively titrated using a SAT antigen (Qingdao Lijian Biological Technology Co., Ltd.; veterinary drug approval number: 兽药生字154028011) in accordance with Chinese national standard GB/T 18646 for Brucellosis Diagnostic Techniques (17, 18). Briefly, the antigen was diluted 1:20, and test sera were prepared in serial dilutions at 1:12.5, 1:25, 1:50, 1:100, and 1:200, with negative and positive serum controls included in parallel. Each tube received 0.5 mL of diluted antigen and 0.5 mL of diluted serum and was incubated at 37 °C for 24 h before reading. In accordance with GB/T 18646 criteria for goats, a SAT titer of 1:50 (++) was designated as the positive threshold, and 1:25 (++) as suspect; suspect animals were retested after 3–4 weeks, and those remaining suspect on retest were classified as positive (16, 17). It should be noted that serological stratification in this study was used to characterize the level of serological reactivity and does not directly equate to a precisely defined infection timeline, bacterial burden, or pathological stage. Based on two-step serological testing, all submitted animals were classified into three serological strata of 50 animals each: seronegative (Group A), defined as RBPT-negative; weakly seropositive (Group B), defined as RBPT-positive with a SAT titer of 1:50 (++); and strongly seropositive (Group C), defined as RBPT-positive with a SAT titer of ≥1:100 (++). From each group of 50 animals, eight were selected for metabolomic analysis after meeting the inclusion criteria: no hemolysis and sufficient serum volume. This selection yielded a total of 24 serum samples. With *n =* 8 animals per serological stratum, the sample size aligns with that adopted in previous discovery-phase livestock serum metabolomics work (11, 14). All results are exploratory in nature and require further validation using larger independent cohorts. All samples were delivered to Novogene Co., Ltd. (Beijing, China) for metabolomic profiling.

### 2.4 Serum metabolomic analysis

#### 2.4.1 Sample preparation

A volume of 100 μL of serum was combined with 400 μL of a methanol/acetonitrile extraction solution (1:1, v/v) containing isotope-labeled internal standards. Samples were vortex-mixed for 3 min, incubated at −20 °C for 30 min to precipitate proteins, and centrifuged at 12,000 r/min for 10 min at 4 °C. The supernatant was collected, re-incubated at −20 °C for an additional 30 min, and centrifuged again at 12,000 r/min for 3 min at 4 °C. A final volume of 180 μL of the resulting supernatant was transferred to autosampler vials and stored at −80 °C until instrument analysis. All sample preparation steps were performed on ice. Quality control (QC) samples were prepared by pooling equal volumes of supernatant from all experimental samples; one QC sample was inserted after every 10 experimental samples throughout the analytical run to monitor the stability of the chromatographic–mass spectrometric system.

#### 2.4.2 Chromatographic and mass spectrometric conditions

Serum metabolic profiling was performed using a broad-targeted LC–MS/MS metabolomics strategy. The liquid chromatography system comprised an ExionLC AD ultra-high-performance liquid chromatography (UHPLC) system (AB SCIEX, Foster City, CA, USA) fitted with an ACQUITY UPLC HSS T3 C18 column (100 mm × 2.1 mm i.d., 1.8 μm; Waters Corporation, Milford, MA, USA). Mobile phase A consisted of 0.1% (v/v) formic acid in water, and mobile phase B consisted of 0.1% (v/v) formic acid in acetonitrile. The gradient elution program was as follows: 0–1 min, 95% A; 1–11 min, linear gradient to 5% A; 11–12 min, held at 5% A; 12–12.1 min, returned to 95% A; 12.1–14 min, held at 95% A for column re-equilibration. The flow rate was 0.40 mL/min, the injection volume was 2 μL, and the column temperature was maintained at 40 °C. Mass spectrometric detection was performed on a SCIEX QTRAP® 6,500 + triple quadrupole/linear ion trap hybrid mass spectrometer (AB SCIEX) equipped with an electrospray ionization (ESI) Turbo Ion-Spray source. Metabolite detection and annotation were based on the Novogene in-house metabolomics database (novoDB), with relative abundance obtained via the LC–MS/MS platform. Following ionization, precursor ions of defined mass-to-charge ratios (m/z) were selected by the Q1 quadrupole and fragmented in the Q2 collision cell at defined collision energies to generate product ions, which were subsequently filtered by the Q3 quadrupole prior to detection. Multiple reaction monitoring (MRM) was applied for all metabolite measurements. ESI source parameters in both positive and negative ion modes were as follows: ion source temperature, 500 °C; ion spray voltage, +5,500 V (positive mode) and −4,500 V (negative mode); ion source gas 1 (GS1), 55 psi; ion source gas 2 (GS2), 60 psi; curtain gas (CUR), 25 psi; collision-activated dissociation (CAD), set to high. Both Q1 and Q3 were operated at unit resolution; the dwell time per MRM ion pair was 3 ms, and the polarity switching time was ≤50 ms.

#### 2.4.3 Metabolite annotation and relative quantification

Metabolite annotation was achieved by matching retention times, precursor/product ion pairs, and secondary mass spectral information against the novoDB database. Raw mass spectrometric data were processed using SCIEX OS software (v1.4; AB SCIEX) for peak extraction, integration, and calibration. The chromatographic peak area of each metabolite served as a proxy for its relative abundance and was exported for downstream statistical analysis. In accordance with the Metabolomics Standards Initiative (MSI) reporting guidelines (19), all metabolite annotations reported herein are classified as MSI Level 2 identifications, based on matching of retention time, precursor ion m/z, and MS/MS fragmentation spectra against the novoDB library, without confirmation by authentic reference standards analyzed under identical instrumental conditions. Level 1 identification, which requires co-elution and co-fragmentation with an authentic reference standard in the same analytical run, was not applied. Accordingly, isomeric ambiguity cannot be excluded for structurally similar compounds, and all metabolite names should be regarded as tentative annotations.

#### 2.4.4 Data preprocessing and quality control

Raw peak area data underwent the following quality control and preprocessing steps prior to statistical analysis. First, Pearson correlation coefficients were calculated for each metabolite across all QC samples; a minimum value of ≥0.90 was required for all QC sample pairs to confirm within-batch reproducibility. Principal component analysis (PCA) was applied to both experimental and QC samples to verify instrument stability, with QC samples expected to cluster tightly in the scores plot. Second, the relative standard deviation (RSD) of each metabolite across QC samples was calculated, and features with RSD > 30% were removed to eliminate systematic error attributable to instrument variation. All QC-based filtering was completed prior to any downstream statistical analysis.

### 2.5 Statistical analysis

All statistical analyses were performed in R (version 4.4.2) (20). Raw peak area data were first subjected to log₁₀ transformation followed by unit variance (UV) scaling to normalize skewed distributions and remove dimensional differences between variables (21). Normalized data were used for all subsequent analyses. Unsupervised visualization was first conducted by principal component analysis (PCA), a dimensionality-reduction method that identifies linear combinations of metabolite variables accounting for maximum variance in the dataset without reference to class labels, to reveal principal patterns of metabolic variation and to assess the latent stratification structure within the data. Supervised discrimination was subsequently performed using partial least squares–discriminant analysis (PLS-DA), a supervised classification algorithm that maximizes the covariance between the metabolite matrix (X) and the binary class indicator matrix (Y) by projecting both into a shared latent space. Model performance was summarized by the in-sample coefficient of determination (R^2^Y) and cross-validated predictive relevance (Q^2^Y). Model robustness was evaluated by 7-fold cross-validation and 1,000 permutation tests, in which class labels were randomly shuffled to generate a null distribution; models were considered valid when the Q^2^ intercept from permutation was below zero (22, 23). Given the limited sample size (*n =* 8 per serological stratum), leave-one-out cross-validation (LOOCV) was additionally performed as the most conservative validation scheme feasible for this design. In LOOCV, each sample was withheld once as the test set while the remaining 15 samples were used to train the model, yielding 16 independent prediction outcomes per pairwise comparison. Univariate comparisons were performed using two-tailed Student’s *t*-tests. Differentially abundant metabolites were identified by the following criteria: variable importance in projection (VIP) > 1.0, fold change (FC) > 1.2 or <0.833, and *p* < 0.05. To control the false-positive rate arising from multiple comparisons, *p*-values were adjusted by the Benjamini–Hochberg (BH) method (21, 24, 25). Differentially abundant metabolites were additionally characterized by hierarchical cluster analysis (Ward. D2 linkage, Euclidean distance), Pearson correlation analysis, Venn diagram comparison, and Kyoto Encyclopedia of Genes and Genomes (KEGG) pathway enrichment analysis to identify metabolite co-variation patterns and associated biological pathways. To complement the primary KEGG enrichment analysis, topology-weighted over-representation analysis (ORA) was performed on KEGG-annotated differentially abundant metabolites for each pairwise comparison. Topology-weighted ORA was conducted using the *Capra hircus* KEGG pathway library (chx), following the MetaboAnalyst framework^1^ (26). Enrichment significance was assessed by a hypergeometric test, and pathway impact was evaluated using a topology-based impact score (relative-betweenness centrality). Receiver operating characteristic (ROC) curve analysis and area under the curve (AUC) were used to evaluate discriminant capacity; metabolites with AUC ≥ 0.70 were considered to exhibit meaningful discriminant performance. Cross-stratum abundance trajectory analysis was additionally performed for metabolites satisfying all three criteria (VIP > 1.0, *p* < 0.05, and AUC ≥ 0.70). Given the limited sample size and absence of an independent validation cohort, all ROC-based evaluations in this study should be interpreted as preliminary and exploratory.

## 3 Results

### 3.1 Metabolomic data quality control

**Figure 1:**
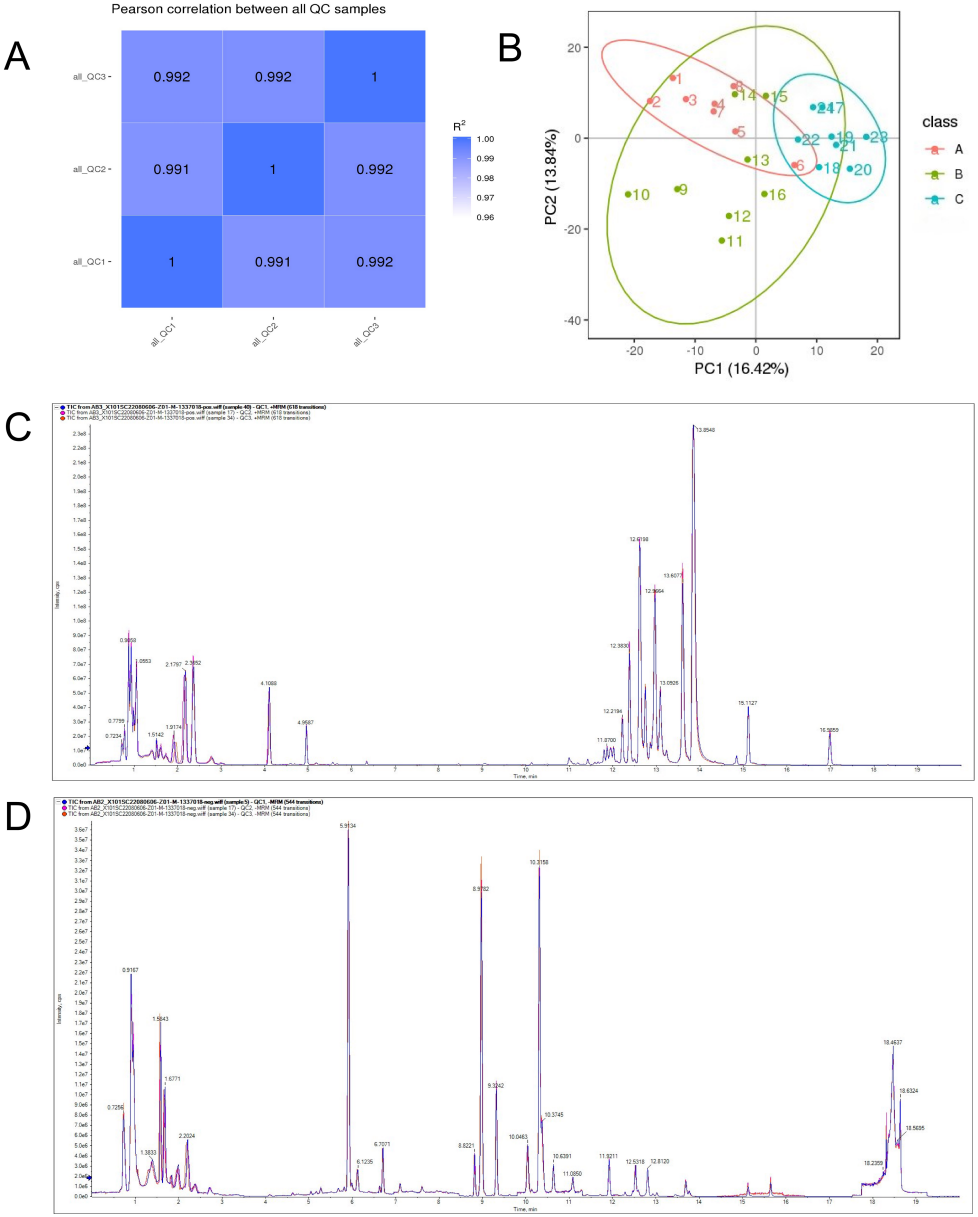
Quality control assessment of metabolomic data. **(A)** Pearson correlation analysis among QC samples; **(B)** PCA scores plot of all experimental and QC samples; **(C,D)** Base peak chromatograms (BPCs) in positive and negative ion modes, respectively. Tight QC sample clustering in the PCA scores plot confirms analytical stability.

Pearson correlation coefficients calculated across all quality control (QC) samples ranged from 0.991 to 1.000, with coefficients of determination (R^2^) approaching 1.0 (Figure 1A), indicating excellent within-batch analytical reproducibility. Principal component analysis (PCA) of all experimental and QC samples showed tight clustering of QC samples in the scores plot, with PC1 and PC2 accounting for 15.91 and 13.14% of total variance, respectively (Figure 1B), confirming instrument stability throughout the run. Base peak chromatograms (BPCs) of QC samples in both positive and negative ion modes displayed highly consistent peak profiles with minimal variation in retention time and signal intensity (Figures 1C,D). Together, these results confirm adequate analytical reproducibility and system stability, validating all subsequent metabolomic analyses.

### 3.2 Metabolite annotation and classification overview

Using the LC–MS/MS platform in conjunction with the Novogene in-house metabolomics database (novoDB), a total of 699 metabolites were annotated and assigned to 39 structural categories. The largest classes included amino acids and their derivatives (*n =* 135), organic acids and their derivatives (*n =* 108), fatty acyls (*n =* 57), nucleotides and their derivatives (*n =* 55), phospholipids (*n =* 31), and bile acids (*n =* 31). A complete list of annotated metabolites is provided in Supplementary Table S1.

### 3.3 Multivariate statistical analysis

#### 3.3.1 Principal component analysis

**Figure 2:**
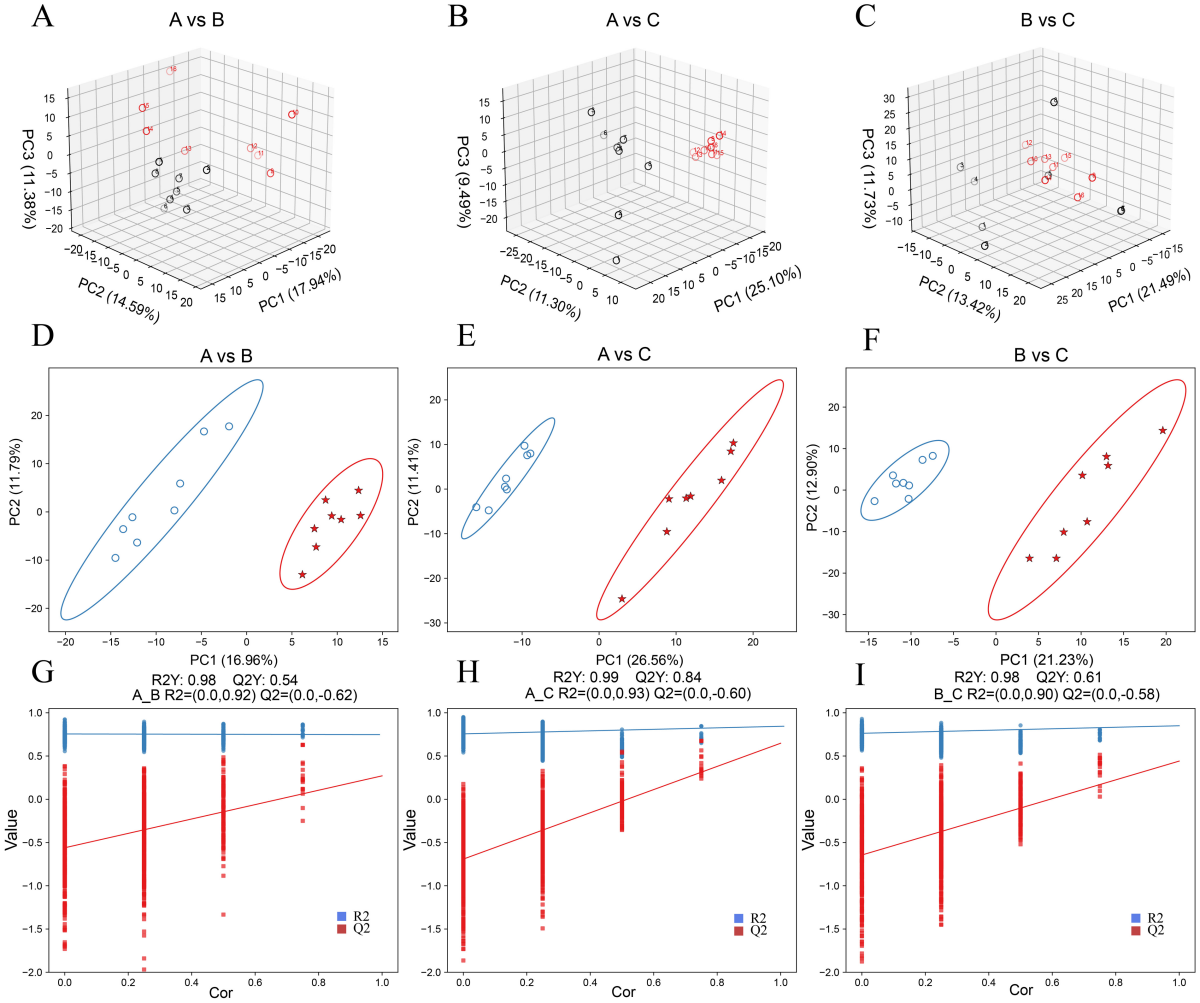
Multivariate statistical analysis of serum metabolic profiles in Saanen goats across serological strata. **(A–C)** PCA scores plots for Group A vs. Group B, Group A vs. Group C, and Group B vs. Group C, respectively; **(D–F)** corresponding PLS-DA scores plots; **(G–I)** permutation validation results (1,000 permutations). Axes represent the first two components with the percentage of explained variance in parentheses. Each point represents one sample; ellipses indicate 95% confidence intervals.

PCA was applied to each pairwise group comparison to provide an unsupervised overview of global metabolic variation across serological strata (Figures 2A–C). In the Group A vs. Group B comparison, PC1 and PC2 explained 17.94 and 14.59% of total variance, respectively (Figure 2A); in Group A vs. Group C, the corresponding values were 25.10 and 11.30% (Figure 2B); and in Group B vs. Group C, 21.49 and 13.42% (Figure 2C). Partial inter-group separation was evident in all three scores plots, with within-group samples showing relative cohesion. Separation was most apparent between Groups A and C. A progressive increase in inter-group separation with increasing serological reactivity (seronegative → weakly seropositive → strongly seropositive) was apparent, suggesting a consistent relationship between metabolic profile divergence and serological stratification.

#### 3.3.2 PLS-DA modeling and permutation validation

To further resolve inter-group metabolic differences and assess potential overfitting, partial least squares-discriminant analysis (PLS-DA) models were constructed for each pairwise comparison (Figures 2D–F). In the Group A vs. Group B comparison, PLS-DA scores demonstrated clear inter-group separation (Figure 2D), with R^2^Y = 0.98 and Q^2^Y = 0.54. In the Group A vs. Group C comparison, separation was more pronounced (Figure 2E), yielding R^2^Y = 0.99 and Q^2^Y = 0.84, indicating the greatest metabolic divergence between seronegative and strongly seropositive animals. In the Group B vs. Group C comparison, R^2^Y = 0.98 and Q^2^Y = 0.61 (Figure 2F), reflecting effective inter-group discrimination. All R^2^Y values approached 1.0, indicating high model fit; Q^2^Y values exceeded 0.50 in all comparisons (reaching 0.84 for Group A vs. Group C), suggesting meaningful predictive relevance. Permutation validation results (1,000 permutations) are presented in Figures 2G–I. In all three models, R^2^ intercepts exceeded Q^2^ intercepts (Group A vs. Group B: R^2^ = 0.92, Q^2^ = −0.62; Group A vs. Group C: R^2^ = 0.93, Q^2^ = −0.60; Group B vs. Group C: R^2^ = 0.90, Q^2^ = −0.58), and Q^2^ regression lines intersected the y-axis below zero in all cases, collectively indicating the absence of model overfitting and confirming adequate statistical reliability. To address potential overfitting associated with the small sample size (*n =* 8 per group), leave-one-out cross-validation (LOOCV) was applied to each pairwise PLS-DA model. LOOCV classification accuracies were 87.5% for A vs. B (14/16 correct), 93.8% for A vs. C (15/16 correct), and 93.8% for B vs. C (15/16 correct), substantially above chance (50%) (Supplementary Figure S1 and Supplementary Table S4). Misclassified samples were predominantly weakly seropositive animals (B14 and B15 in A vs. B; A6 in A vs. C; B15 in B vs. C), consistent with gradation along the serological continuum rather than random misclassification.

### 3.4 Identification and global distribution of differentially abundant metabolites

**Figure 3:**
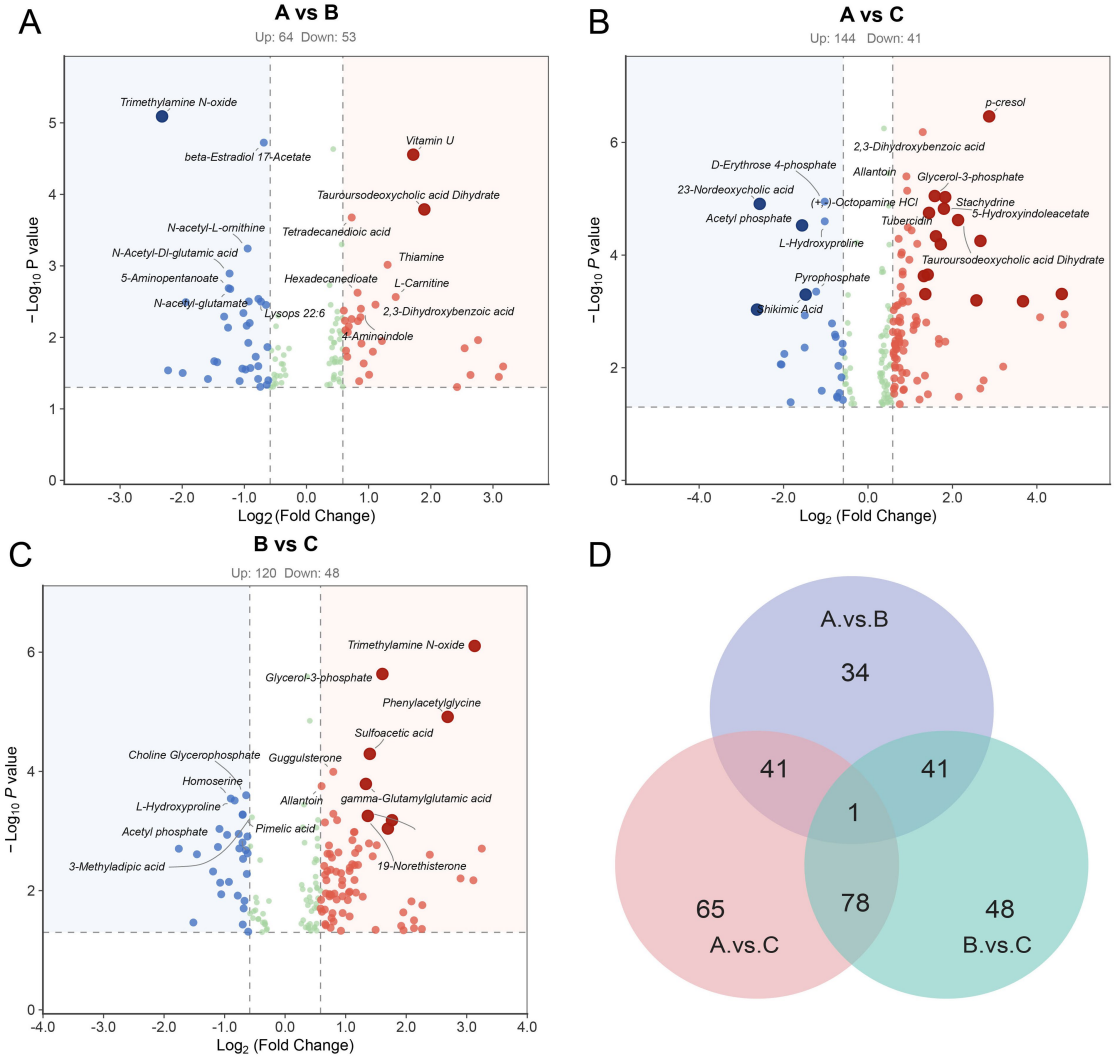
Identification and distribution of differentially abundant metabolites across serological strata. **(A–C)** Volcano plots for Group A vs. Group B, Group A vs. Group C, and Group B vs. Group C comparisons, respectively. Red and blue points indicate significantly upregulated and downregulated metabolites, respectively; **(D)** Venn diagram showing the overlap of differentially abundant metabolites across the three pairwise comparisons.

Applying the predefined screening criteria (variable importance in projection (VIP) > 1.0, fold change (FC) > 1.2 or <0.833, and *p* < 0.05), differentially abundant metabolites were identified in each pairwise comparison: 117 in Group A vs. Group B (64 upregulated, 53 downregulated), 185 in Group A vs. Group C (144 upregulated, 41 downregulated), and 168 in Group B vs. Group C (120 upregulated, 48 downregulated) (Figures 3A–C; Supplementary Table S2). The greatest number of differentially abundant metabolites was observed in the Group A vs. Group C comparison, consistent with the most pronounced metabolic divergence identified in the multivariate analyses. Cross-comparison Venn diagram analysis revealed that D-erythrose 4-phosphate was the only metabolite significantly altered in all three pairwise comparisons (Figure 3D), identifying it as a candidate metabolite with consistent association across serological strata.

### 3.5 Hierarchical clustering and Pearson correlation analysis

**Figure 4:**
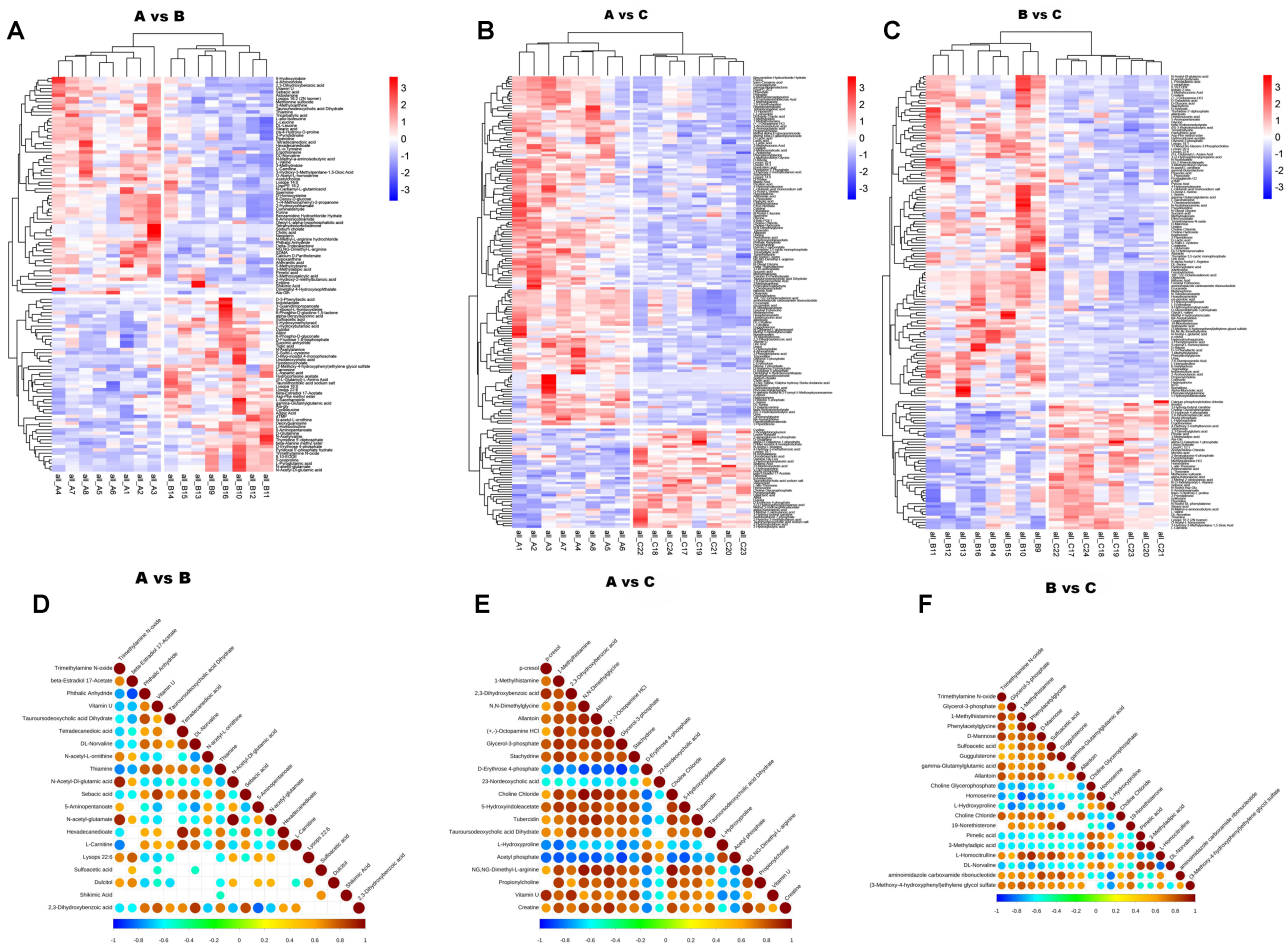
Expression patterns and correlation structure of differentially abundant metabolites. **(A–C)** Hierarchical clustering heatmaps for Group A vs. Group B, Group A vs. Group C, and Group B vs. Group C, respectively; **(D–F)** Pearson correlation heatmaps of the corresponding differentially abundant metabolites. Color scales indicate metabolite relative abundance (Z-score) and Pearson correlation coefficient, respectively.

To characterize the expression patterns of differentially abundant metabolites, hierarchical clustering was performed using the Ward. D2 linkage method and Euclidean distance. In all three pairwise comparisons, samples clustered clearly by serological stratum, with high within-group homogeneity (Figures 4A–C). Comparisons involving Group C yielded a greater number of metabolite clusters than the Group A vs. Group B comparison, reflecting the larger magnitude of metabolic change associated with strongly seropositive status. Pearson correlation analysis of differentially abundant metabolites revealed that both the number of significantly correlated metabolite pairs and the strength of pairwise correlations increased progressively in comparisons involving Group C (Figures 4D–F). Overall, metabolite co-variation networks became increasingly complex with rising serological reactivity, suggesting more extensive coordinated metabolic regulation at higher serological reactivity levels.

### 3.6 Abundance trajectory patterns of candidate metabolites across serological strata

**Figure 5:**
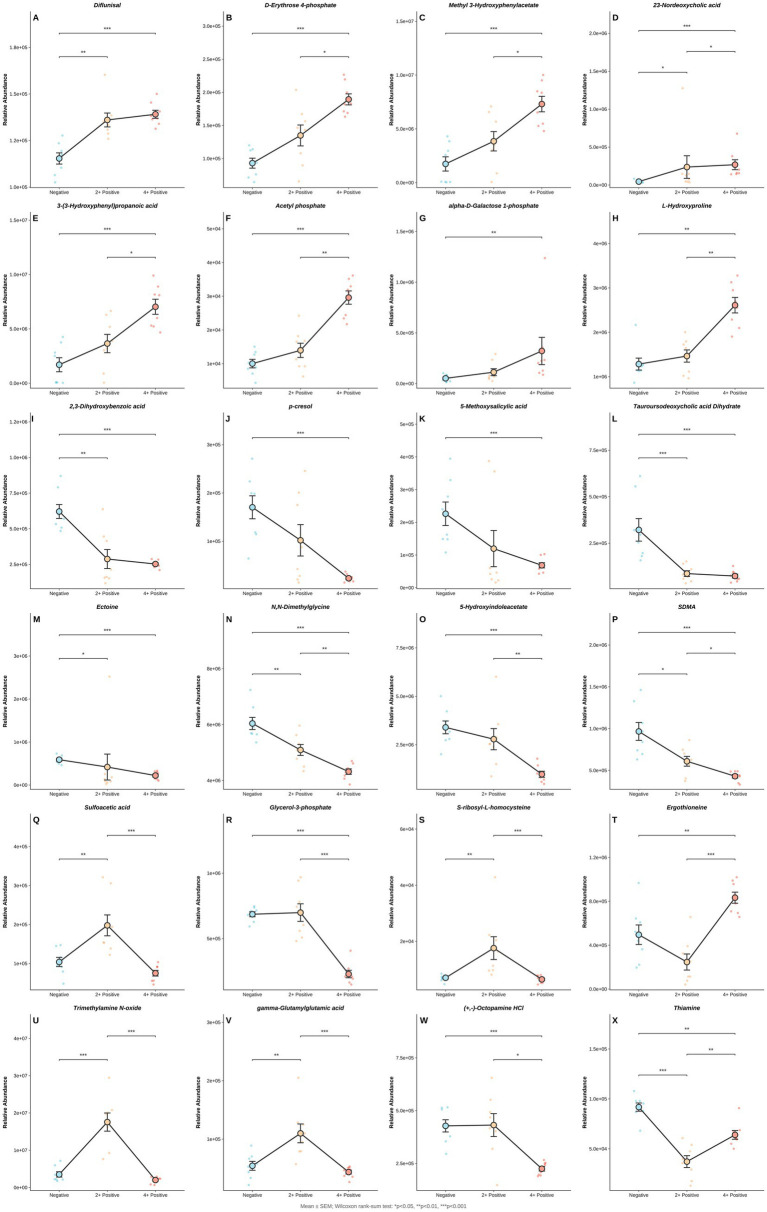
Abundance trajectory patterns of representative candidate metabolites across serological strata in Saanen goats. **(A–H)** Metabolites with monotonically increasing abundance; **(I–P)** metabolites with monotonically decreasing abundance; **(Q–X)** metabolites with non-linear abundance patterns. Metabolites were selected on the basis of VIP > 1.0, *p* < 0.05, and AUC ≥ 0.70. Individual sample values are shown as scatter points; connecting lines indicate group means; error bars represent the standard error of the mean (SEM).

To characterize how metabolite abundances varied across serological strata, representative metabolites were selected based on VIP > 1.0, *p* < 0.05, and area under the receiver operating characteristic curve (AUC) ≥ 0.70 and examined for cross-stratum abundance patterns. As this study employed a cross-sectional design, the term “trajectory” refers to inter-group abundance patterns rather than individual longitudinal changes. Three characteristic pattern types were identified (Figure 5). The first pattern comprised metabolites exhibiting monotonically increasing abundance with serological reactivity. Among these, D-erythrose 4-phosphate was significantly altered in all three pairwise comparisons (Group A vs. Group B: AUC = 0.766; Group A vs. Group C: AUC = 1.000; Group B vs. Group C: AUC = 0.891), demonstrating the most consistent association across serological strata (Figures 5A–H). The second pattern comprised metabolites showing monotonically decreasing abundance. Symmetric dimethylarginine (SDMA) and p-cresol were representative of this group, exhibiting progressive decline from seronegative to strongly seropositive animals (Figures 5I–P). The third pattern encompassed metabolites displaying non-linear changes. Trimethylamine N-oxide (TMAO) exemplified a biphasic pattern, increasing from Group A to Group B before declining in Group C. Additional metabolites displayed inverted U-shaped (peak in Group B) or U-shaped (nadir in Group B) profiles, or showed pronounced changes only at the strongly seropositive stage (Figures 5Q–X).

### 3.7 KEGG pathway enrichment analysis

**Figure 6:**
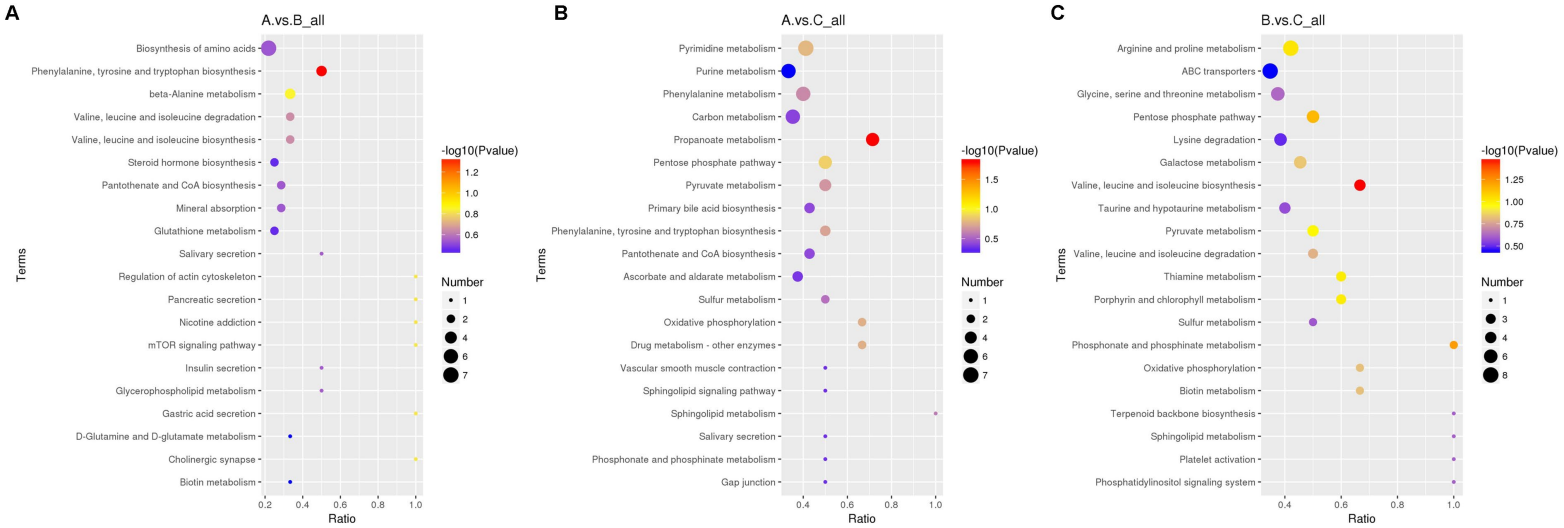
KEGG pathway enrichment analysis of differentially abundant metabolites. **(A)** Group A vs. Group B; **(B)** Group A vs. Group C; **(C)** Group B vs. Group C. The x-axis represents the enrichment ratio; the y-axis lists pathway names. Bubble size reflects the number of metabolites enriched per pathway; color scale indicates −log₁₀(*p*-value).

Kyoto Encyclopedia of Genes and Genomes (KEGG) pathway enrichment analysis of differentially abundant metabolites (Supplementary Table S3) revealed two distinct enrichment patterns across serological strata (Figure 6). Amino acid metabolism pathways—including phenylalanine, tyrosine, and tryptophan biosynthesis as well as branched-chain amino acid (BCAA) metabolism—were consistently and significantly enriched across all three pairwise comparisons. In contrast, energy and nucleotide metabolism pathways—including pyruvate metabolism, the pentose phosphate pathway (PPP), oxidative phosphorylation, and purine/pyrimidine metabolism—were significantly enriched predominantly in comparisons involving Group C, suggesting that perturbations in these pathways are associated with higher levels of serological reactivity. Topology-weighted ORA based on the *Capra hircus* (chx) KEGG library corroborated these primary enrichment patterns (Supplementary Table S5 and Supplementary Figure S2): amino acid metabolism pathways remained among the top-ranked pathways in all comparisons, whereas energy- and nucleotide-related pathways were most prominently represented in comparisons involving Group C.

### 3.8 Exploratory assessment of the discriminant potential of candidate metabolites

**Figure 7:**
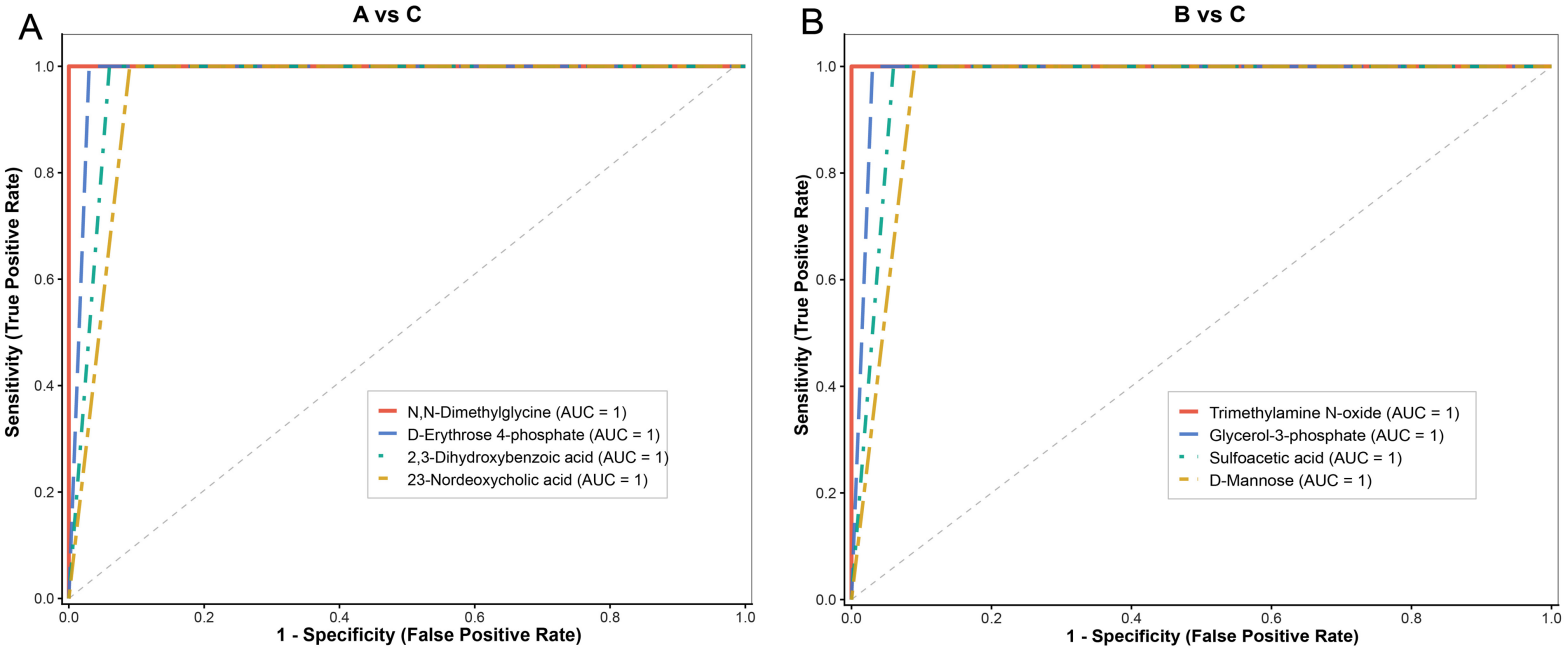
ROC curve analysis of candidate discriminant metabolites. **(A)** Discrimination between Group A (seronegative) and Group C (strongly seropositive); **(B)** Discrimination between Group B (weakly seropositive) and Group C (strongly seropositive). The diagonal dashed line represents the reference baseline for random classification (AUC = 0.500).

Given the limited sample size and absence of an independent validation cohort, the discriminant potential of candidate metabolites was evaluated within the present dataset as a strictly exploratory exercise. Receiver operating characteristic (ROC) curve analyses were conducted for two scenarios: distinguishing seronegative from strongly seropositive animals, and distinguishing weakly from strongly seropositive animals (Figure 7). For the discrimination of strongly seropositive status (Group A vs. Group C; Figure 7A), several metabolites demonstrated high within-dataset discriminant performance, with N, N-dimethylglycine, D-erythrose 4-phosphate, 2,3-dihydroxybenzoic acid, and 23-nordeoxycholic acid each achieving AUC = 1.000. For the discrimination between adjacent serological strata (Group B vs. Group C; Figure 7B), TMAO, glycerol-3-phosphate, sulfoacetic acid, and D-mannose likewise demonstrated high discriminant capacity (AUC = 1.000). Notably, TMAO—which displayed a biphasic abundance trajectory—also showed strong inter-stratum discriminant performance. As the present findings are based on a limited sample, all ROC-derived results require validation in larger, independent cohorts before any diagnostic implications can be drawn.

## 4 Discussion

Goat brucellosis caused by *Brucella melitensis* constitutes the leading source of human brucellosis globally, yet systematic investigation of host metabolic responses across different infection states within naturally exposed populations remains limited (27). The present study characterized serum metabolic profiles associated with distinct Brucella serological strata in Saanen goats sampled under a natural surveillance context. Observable metabolic differences were identified among seronegative, weakly seropositive, and strongly seropositive animals, with the most pronounced divergence occurring between Groups A and C. Compared with conventional binary seropositive/seronegative designs, a serological stratification approach allows more granular characterization of metabolic variation patterns associated with differing levels of serological reactivity (14). We emphasize that the serological strata in this study reflect the intensity of serological reactivity and should not be interpreted as equivalent to precisely defined infection timelines, bacterial burden levels, or pathological stages; the findings are accordingly discussed as serological stratum-associated metabolic reprogramming characteristics.

KEGG pathway enrichment analysis revealed two distinct enrichment patterns, offering insight into the potential temporal sequence of metabolic reorganization during infection. Phenylalanine, tyrosine, and tryptophan biosynthesis, as well as branched-chain amino acid (BCAA) metabolism, were consistently and significantly enriched across all three pairwise comparisons, indicating that amino acid metabolism disruption is a persistent feature shared across serological strata and may reflect the sustained nutritional demands of Brucella for amino acids to support intracellular replication (28, 29). Notably, examination of the enrichment results revealed that both amino acid biosynthesis pathways (e.g., valine, leucine and isoleucine biosynthesis; phenylalanine, tyrosine and tryptophan biosynthesis) and catabolic pathways (e.g., valine, leucine and isoleucine degradation) were concurrently represented across pairwise comparisons, indicating a bidirectional perturbation of the host amino acid pool rather than a unidirectional shift: pathogen-driven catabolic depletion of host amino acid substrates and host compensatory upregulation of biosynthetic pathways appear to operate in parallel (29). In contrast, pyruvate metabolism, the pentose phosphate pathway (PPP), oxidative phosphorylation, and purine/pyrimidine metabolism were significantly enriched only in comparisons involving Group C. This finding is consistent with prior reports demonstrating that *B. abortus* infection induces a Warburg-like metabolic shift in host cells (9) and that *Brucella* infection can upregulate host purine biosynthesis pathways to sustain intracellular proliferation (10, 30). The stage-associated enrichment of energy and nucleotide metabolism pathways therefore suggests that these changes may represent the combined effect of host immunometabolic activation and elevated pathogen metabolic demands at higher serological reactivity levels (30, 31). The coexistence of “persistently enriched” amino acid pathways and “stage-associated” energy and nucleotide pathways implies that these two categories of metabolic change fulfill functionally distinct roles in *Brucella*-associated host responses. These pathway-level patterns were further supported by topology-weighted ORA using the *Capra hircus* (chx) KEGG library, which independently highlighted persistent amino acid metabolism disruption across serological strata and selective enrichment of energy- and nucleotide-related pathways in comparisons involving strongly seropositive animals.

At the level of individual metabolites, D-erythrose 4-phosphate (E4P) was the sole metabolite significantly altered across all three pairwise comparisons, exhibiting a monotonically increasing abundance pattern and progressively stronger discriminant capacity with serological reactivity (Group A vs. Group B: AUC = 0.766; Group A vs. Group C: AUC = 1.000; Group B vs. Group C: AUC = 0.891). The biological significance of E4P is supported by two independent lines of evidence. As a key intermediate of the pentose phosphate pathway (PPP), elevated serum E4P may reflect increased overall PPP flux in *Brucella*-infected and immune-activated host cells. When PPP activity is upregulated, its oxidative branch generates additional NADPH—the obligate substrate for NADPH oxidase (NOX2)-dependent superoxide production and oxidative burst, a central innate antimicrobial mechanism against intracellular *Brucella* (31)— while its non-oxidative branch simultaneously produces E4P as a parallel metabolic output. These two outcomes are concurrent consequences of enhanced PPP flux rather than sequential steps. This host-side interpretation is consistent with the Warburg-like metabolic shift that *Brucella* induces in host cells (9), which characteristically diverts glucose carbon flux toward glycolytic and PPP pathways, thereby supporting both NADPH regeneration and the accumulation of non-oxidative branch intermediates including E4P. Concurrently, Barbier et al. (28) demonstrated that *Brucella* can catabolize erythritol to yield E4P, which subsequently enters the shikimate pathway to support the biosynthesis of aromatic amino acids and the iron-chelating siderophore 2,3-dihydroxybenzoic acid. In the present study, 2,3-dihydroxybenzoic acid also exhibited high discriminant capacity (AUC = 1.000 for Group A vs. Group C), providing additional support for this metabolic linkage. Whether the rise in E4P is driven primarily by host PPP activation, by *Brucella*-side erythritol catabolism, or by both acting in concert cannot be resolved from the present data; isotope tracing experiments will be needed to partition these contributions.

TMAO, a product of gut microbiota-mediated metabolism of choline and related substrates, is closely associated with systemic inflammatory regulation and immunometabolic status (32). In this study, TMAO exhibited a non-linear, biphasic abundance pattern—increasing from Group A to Group B before declining in Group C—suggesting that it may reflect metabolic rearrangements specific to particular serological states rather than a simple monotonic relationship with serological reactivity. By contrast, SDMA declined monotonically across serological strata, a direction opposite to the markedly elevated SDMA levels reported in sepsis patients (33). This divergence suggests that animals at different serological strata may operate under distinct metabolic regulatory states, and that individual metabolites capture different biological dimensions of infection. Together, the contrasting trajectories of TMAO and SDMA illustrate that no single biomarker is likely to comprehensively reflect the complexity of the infection-associated metabolome.

The progressive increase in metabolite co-variation complexity with serological reactivity suggests that *Brucella* infection does not perturb metabolism in isolated pockets but instead triggers a coordinated, network-wide response—a pattern consistent with the broad immunometabolic remodeling documented in prior studies (34).

From a translational perspective, the differentially abundant metabolites identified in this study fall into three functional categories based on their abundance trajectories. Monotonically changing metabolites—exemplified by E4P—show potential as stratum-associated candidate indicators within the present dataset. Biphasic metabolites such as TMAO may be informative for identifying transitional states between specific serological strata. Metabolites that change specifically in the strongly seropositive group represent potential candidate indicators for high-reactivity serological states. The actual diagnostic utility of any of these metabolites, however, is contingent on validation in independent cohorts with substantially larger sample sizes.

Several limitations of this study warrant consideration. First, the metabolomics analysis included only eight animals per serological stratum, which limits statistical power and the generalizability of multivariate models. Although PLS-DA permutation tests yielded Q^2^ intercepts below zero and leave-one-out cross-validation (LOOCV) supported classification above chance, high in-sample R^2^Y values nevertheless warrant cautious interpretation; all multivariate and biomarker findings require replication in larger independent cohorts. Second, the serological stratification design precludes direct interpretation of group differences as representing discrete infection stages, bacterial burden levels, or causal transitions. Third, metabolite annotations were classified as MSI Level 2 identifications based on database matching without confirmation by authentic reference standards; isomeric ambiguity cannot be excluded, and certain metabolites of exogenous or environmental origin may be influenced by non-disease factors—biological interpretations should therefore be treated with appropriate caution. Despite these limitations, this study provides exploratory evidence that *Brucella* infection is accompanied by dynamic, stratum-dependent metabolic reprogramming characterized by persistent disruption of amino acid metabolism and stage-associated intensification of energy metabolism, and identifies candidate metabolites warranting further investigation in adequately powered validation studies.

## 5 Conclusion

This study characterized the serum metabolic perturbations associated with *Brucella* serological reactivity in Saanen goats sampled under a natural infection surveillance context, using broad-targeted LC–MS/MS serum metabolomics. Serum metabolic profiles differed detectably across the three serological strata, with the greatest separation between seronegative and strongly seropositive animals. Amino acid metabolism was disrupted regardless of reactivity level, whereas perturbations in energy and nucleotide metabolism emerged specifically in the strongly seropositive group. D-erythrose 4-phosphate, trimethylamine N-oxide, and symmetric dimethylarginine exhibited serological stratum-associated abundance patterns and demonstrated exploratory discriminant potential within the present dataset.

Collectively, these findings provide exploratory evidence for serological stratum-dependent metabolic reprogramming associated with *Brucella* infection in goats, and offer a set of candidate metabolites for prioritization in future studies employing larger sample sizes and independent validation cohorts. The stratification approach used here may also prove useful in other naturally exposed livestock species where binary serology fails to capture the full spectrum of host responses.

## Data Availability

The data presented in the study are deposited in the Figshare repository under DOI: https://doi.org/10.6084/m9.figshare.32686290.

## Footnotes

1
www.metaboanalyst.ca
