# Supplementary figures and images for: Stratum-specific serum metabolic reprogramming in Saanen goats with graded Brucella serological reactivity under natural exposure
Source: Front Vet Sci. 2026 Jun 26;13:1866534. doi: 10.3389/fvets.2026.1866534 (PMC13354091; doi:10.3389/fvets.2026.1866534)

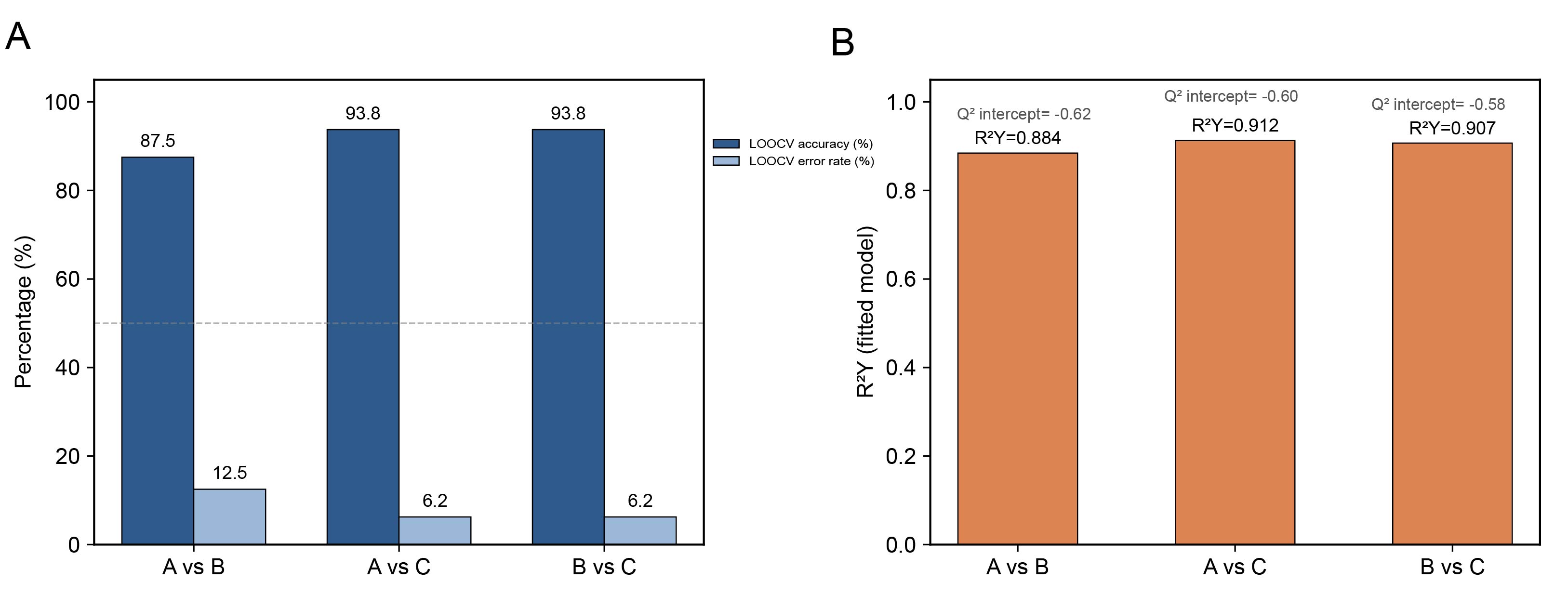

Supplement: Supplementary file 1 [file Image_1.JPEG]

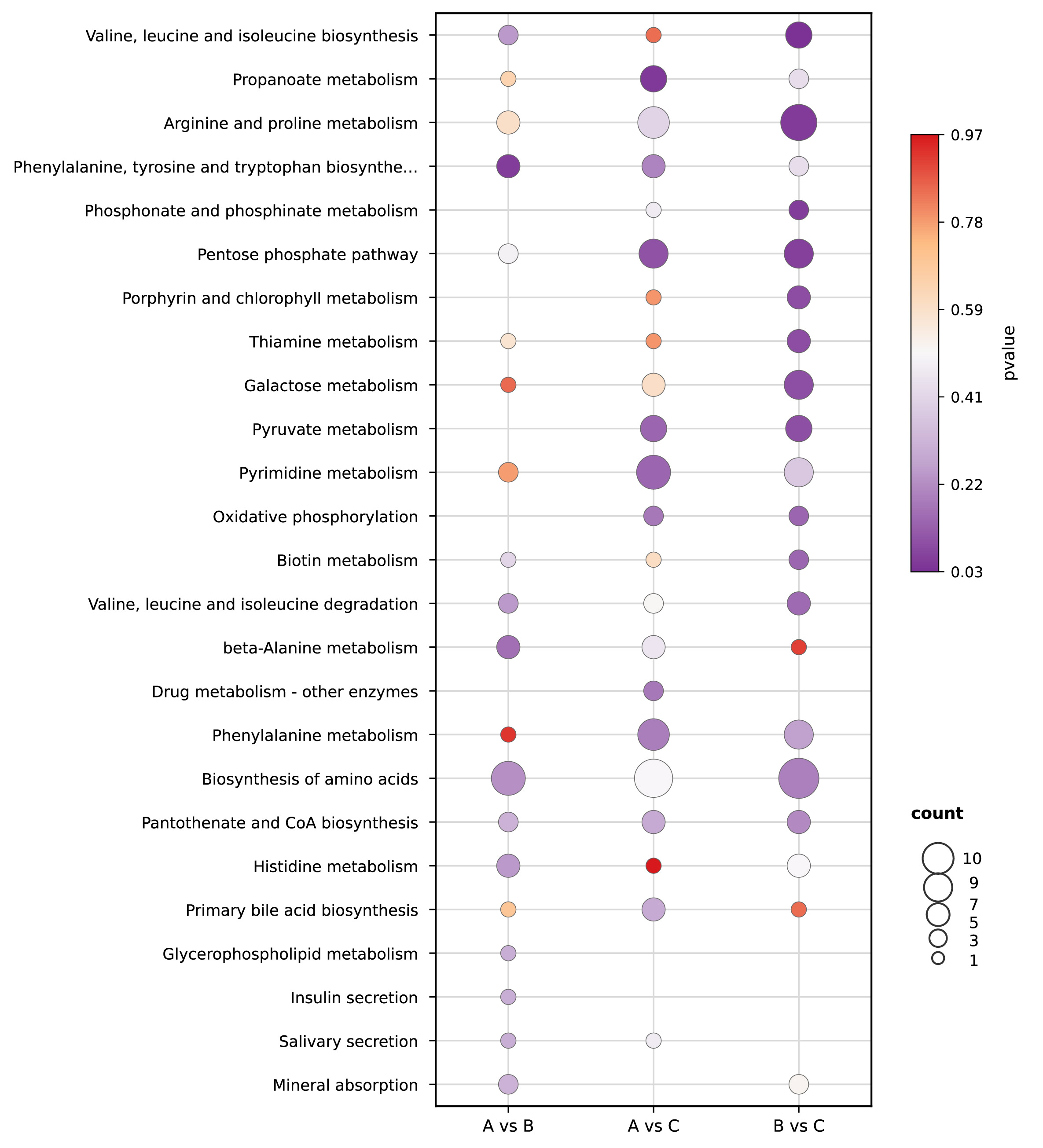

Supplement: Supplementary file 2 [file Image_2.JPEG]
